# Concordance of the Fracture Risk Assessment Tool With and Without Bone Mineral Density and Its Role in Predicting Postoperative Proximal Junctional Kyphosis After Adult Spinal Deformity Surgery

**DOI:** 10.1227/neuprac.0000000000000182

**Published:** 2025-10-20

**Authors:** Junya Katayanagi, Hiroyuki Inose, Tomoyuki Tanaka, Hiroki Konuma, Tsukasa Yanase, Takahiro Iida, Shingo Morishita, Tetsuya Jinno

**Affiliations:** 1 Department of Orthopedic Surgery, Dokkyo Medical University Saitama Medical Center, Koshigaya, Saitama, Japan;; 2 Department of Orthopedic Surgery, Teine Keijinnkai Hospital, Sapporo, Hokkaido, Japan;; 3 Department of Orthopedic Surgery, Tokyo Bay Urayasu Ichikawa Medical Center, Urayasu, Chiba, Japan

**Keywords:** Fracture risk assessment tool, Proximal junctional kyphosis, Adult spinal deformity, Surgery, Bone density

## Abstract

**BACKGROUND AND OBJECTIVES:**

To compare the 10-year risk of major osteoporotic fracture (MOF) without bone density (MOF-BD) data and MOF with BD (MOF + BD) data to determine their validity as risk prediction tools for postoperative proximal junctional kyphosis (PJK) with vertebral fracture after adult spinal deformity (ASD) surgery. ASD surgery can cause mechanical complications, such as PJK. Osteoporosis is linked to PJK; therefore, predicting bone fractures by BD is important. The fracture risk assessment tool was used to assess fracture risk, and the addition of BD information might have increased its accuracy.

**METHODS:**

This study obtained data from medical records, surgical factors, spinal alignment data, and imaging of patients with ASD.

**RESULTS:**

Ninety-two patients were enrolled in the study (mean age at surgery: 67.8 years; mean follow-up: 8.0 years). A comparison of fracture risk assessment tool calculations in ASD patients demonstrated excellent agreement (r = 0.866; 95% CI: 0.801-0.910; Spearman correlation coefficient) for major fractures between the MOF-BD and MOF + BD groups. Independent risk factors for PJK with fracture after ASD surgery included baseline pelvic tilt (>30°), pelvic tilt at first postoperative standing (>30°), and MOF-BD (>15%). Age (r = 0.400, *P* < .001), prior fragility fracture (r = 0.467, *P* < .001), and secondary osteoporosis (r = 0.388, *P* < .001) were moderately correlated with dissociation between MOF-BD and MOF + BD (all Spearman correlation coefficient). No significant difference in the cumulative hazard of MOF + BD and MOF-BD was observed (*P* = .685; log-rank test).

**CONCLUSION:**

The simpler model of 10-year risk of MOF without BD was in good agreement with BD and may aid general health screening as well as preoperative risk assessment for spinal reconstructive surgery.

**ABBREVIATIONS:**
ASD: adult spinal deformity
BD: bone density
DXA: dual-energy X-ray absorptiometry
FRAX: fracture risk assessment tool
HR: hazard ratio
ICC: intraclass correlation coefficient
L1PA: L1 pelvic angle
LIV: lower instrumented vertebra
LL: lumbar lordosis
MOF: major osteoporotic fracture
PI−LL: pelvic incidence minus LL
PJK: proximal junctional kyphosis
PT: pelvic tilt
QCT: quantitative computed tomography
SRS: Scoliosis Research Society
SVA: sagittal vertical axis
UIV: upper instrumented vertebra
VF: vertebral fracture.

Owing to the aging of society and advances in medical technology, adult spinal deformity (ASD) surgery has become widely used, providing good clinical outcomes for many patients.^1,2^ However, mechanical complications, such as proximal junctional kyphosis (PJK), which is a serious complication after spinal deformity surgery in adults, occur as frequently as 30%-50%, resulting in pain, progressive deformity, and sometimes spinal paralysis.^3-5^ Factors contributing to PJK include overcorrection, long fixation range including the pelvis, and inappropriate rod geometry, which are being addressed through research on sagittal spine alignment and innovations in spinal instrumentation technology.^6-11^ However, the issue of osteoporosis, a patient factor in PJK, remains unresolved and a subject of debate.^12,13^ Many methods are used to measure bone density (BD) when assessing bone health or osteoporosis, including dual-energy X-ray absorptiometry (DXA), quantitative computed tomography (QCT), and computed tomography (CT) values of vertebral bodies and existing vertebral fractures (VFs), which are highly recommended by expert panelists in spinal reconstructive surgery.^14-24^ Although each modality has a certain level of diagnostic performance and is useful in clinical practice, DXA, QCT, and CT values are difficult to apply to all patients when considering the facilities of medical institutions, medical costs, and additional radiation exposure to patients.^15,25,26^ By contrast, the fracture risk assessment tool (FRAX) is relatively simple and commonly used in osteoporosis management to assess fracture risk.^27^ FRAX has been validated in several countries, with local studies and epidemiological data demonstrating its clinical applicability in different populations.^28^ This study aimed to predict the 10-year risk of major osteoporotic fracture (MOF) associated with osteoporosis. The algorithm is based on an individual analysis of each patient to correlate various risk factors, including age, sex, body mass index (BMI), history of fragility fractures, family history of hip fracture, smoking, prolonged use of corticosteroids, rheumatoid arthritis, other causes of secondary osteoporosis, and high alcohol consumption. Fracture prediction based on these clinical data is clinically useful, but the addition of BD information may increase its accuracy.^28,29^

Fracture prediction by FRAX is also useful for complications in spinal reconstructive surgery,^24^ with an MOF > 15% by FRAX being a risk factor for postoperative PJK with VF in ASD surgery.^30^ However, the concordance between fracture prediction with and without BD information for patients undergoing ASD surgery has not been adequately verified.

This study investigated the concordance between MOF without BD (MOF-BD) and MOF with BD (MOF + BD), clarifying the validity of each as a risk prediction tool for postoperative PJK with VF after ASD surgery.

## METHODS

This retrospective observational study was based on medical records and imaging data used in routine medical care with no invasive procedures or tests. All the patients provided written informed consent to participate in the study. Personal patient information and privacy were protected during the preparation of this study. This study was approved by the Institutional Review Board (Bioethics Committee Dokkyo Medical University Saitama Medical Center) of our institution (approval number: 23018).

### Inclusion and Exclusion Criteria

This review used the medical records of patients with ASD who underwent corrective surgery at our institution. Inclusion criteria were as follows: ASD patient aged 50 years or older at the time of surgery, abnormal radiographic variables (Cobb angle ≥ 20°, sagittal vertical axis [SVA] ≥ 4 cm, or pelvic tilt [PT] ≥ 30°), undergoing corrective long spinal fusion of 6 or more spinal segments, and postoperative follow-up of ≥2 years. Patients with spinal deformities associated with Parkinson disease, Parkinson syndrome, syndromic scoliosis, neuromuscular disease, tumors, or fractures caused by high-energy traumas were excluded. Finally, 92 patients (2 male and 90 female) who fulfilled the above criteria were enrolled in the study. The mean age at surgery was 67.8 years (range: 50-86 years), and the mean follow-up was 8.0 years.

### Data Acquisition

A medical record review was conducted, and comprehensive patient information, including age, weight, height, family history of hip fracture in parents, current smoking status, use of glucocorticoids, presence of rheumatoid arthritis, causes of secondary osteoporosis, and alcohol consumption, was extracted. Baseline spinal alignment data encompassed the Cobb angle and lumbar lordosis (LL), as well as 3 sagittal parameters of the Scoliosis Research Society (SRS)-Schwab ASD classification^31^: PT, SVA, pelvic incidence minus LL (PI−LL), L1 pelvic angle (L1PA), and T4 pelvic angle. Furthermore, these radiographic measurements were recorded at the time of immediate postoperative standing and at the final follow-up (over 2 years). BD at the femoral neck was measured using DXA and femoral neck T-score, and the 10-year probability of MOF was calculated using the FRAX tool. In addition, osteoporosis medications administered for at least 3 months before ASD surgery were documented. Surgical factors included changes in the LL angle at first standing after surgery, as well as levels of the upper instrumented vertebra (UIV) and lower instrumented vertebra (LIV), osteotomy grade of the lumbar vertebral column, lateral lumbar interbody fusion, screw density, and UIV fixation type (pedicle screw or transverse process hook). Lumbar osteotomy was graded according to the classification by Schwab et al.^32^ Screw density was calculated by taking the total number of posterior screws placed and divided it by the number of fused vertebral segments.

### Definition of PJK With VF

The incidence of postoperative PJK was assessed according to the definition by Glattes et al,^33^ with PJK accompanied by VF categorized as Yagi-Boachie type 2 (bone failure).^34^ Radiographic evaluations were scheduled at 1, 3, 6, and 12 months after surgery and annually thereafter, with additional imaging performed if clinically indicated (e.g., severe back pain or progression of deformity).

### Statistical Analysis

To assess the agreement between MOF + BD and MOF-BD in this population, Spearman correlation analysis and intraclass correlation coefficient (ICC) were performed by comparing the distribution of data from both groups. In addition, a Brand-Altman plot was generated to visually assess agreement between the 2 data sets. The factors affecting the dissociation of these 2 data sets were identified using correlation analysis. A Cox proportional hazards model with both data sets as covariates was performed to evaluate the effect on postoperative PJK with VF, followed by survival analysis. Covariates included age, BMI, fracture probability according to FRAX (MOF-BD or MOF + BD >15% vs ≤15%), UIV level, LIV level, changes in LL, osteotomy grade at lumbar, UIV fixation type, 3 sagittal parameters of the SRS-Schwab ASD classification at baseline and at first standing, and previous bone-forming treatment for osteoporosis. Variance inflation factors were calculated using logistic regression analysis to confirm multicollinearity. Furthermore, Kaplan-Meier curves for the hazard function of PJK with VF were plotted for MOF-BD and MOF + BD, and the cumulative hazard was compared (log-rank test). Receiver operating characteristic analysis was performed to evaluate the predictive ability of MOF + BD and MOF-BD for PJK occurrence. Because of the large sex bias in this population (male individuals accounted for 2.2% of all patients), we added a similar analysis using a population without male individuals. Data were analyzed using the SPSS 29.0.2.0 (IBM), with a significance level of *P* < .05, using 2-tailed tests.

## RESULTS

### Summary of Baseline Data and Surgical Intervention Factors

Table 1 presents the age, BMI, BD at the femoral neck using DXA, MOF according to FRAX with or without BD (MOF-BD and MOF + BD), surgical intervention factors, and previous treatment for osteoporosis. The mean BD of the femoral neck at baseline was 0.574 g/cm^2^ with a mean T-score of −2.0. The breakdown of risk factors in the FRAX was as follows: family history (parents) of hip fracture in 0 cases, current smoking in 4 cases, glucocorticoids in 1 case, rheumatoid arthritis in 6 cases, causes of secondary osteoporosis in 17 cases, and alcohol consumption in 1 case. The mean baseline MOF-BD (FRAX score without BD) was 14.5%, and 40.2% of the patients had a probability of MOF-BD >15%. The mean baseline MOF + BD (FRAX score with BD) was 12.8%, and 32.6% of the patients had a probability of MOF + BD >15%.

**TABLE 1.** Summary of Baseline Data, Risk Factors in FRAX, Factors Related to Surgical Intervention, Osteoporosis Treatment, and Surgical Correction

Variable	Baseline
Age, y	67.8 ± 8.0
Younger than 70 y (yes/no), %	53.5 (43:49)
Men:women, %	2.2 (2:90)
BMI, kg/m^2^	22.2 ± 3.6
Over 25 kg/m^2^ (yes/no), %	22.7 (19:73)
BD of femoral neck, g/cm^2^	0.574 ± 0.10
T-score	−2.0 ± 0.9
T-score ≤−2.5:>−2.5, %	38 (35:57)
FRAX	
MOF-BD, %	14.5 ± 10.1
MOF-BD >15:≤15, %	40.2 (37:55)
MOF + BD, %	12.8 ± 7.9
MOF + BD >15:≤15, %	32.6 (30:62)
Risk factor in FRAX, %	
Prior fragility fracture	23 (25)
Parental hip fracture	0 (0.0)
Current smoking	4 (4.3)
Systemic glucocorticoid use	1 (1.1)
Rheumatoid arthritis	6 (6.5)
Secondary osteoporosis	17 (18.5)
Alcohol, 3 or more units/d	1 (1.1)
Factors related to surgical correction, %	
Change in LL (°) over 40° (yes/no)	38.0 (35:57)
UIV, caudal to T10 (yes/no)	80.4 (74:18)
LIV, caudal to S1 (yes/no)	72.8 (67:25)
No. of fusion segments, 8 or more	71.7
Osteotomy grade (≤2:≥3)	79:13
Lateral lumbar interbody fusion	66.3
Screw density	1.75
Construct at UIV (pedicle screw: transverse process hook)	71:21
Previous treatment for osteoporosis (yes/no), %	56.5 (52:40)
Previous treatment by bone forming agent for osteoporosis (yes/no), %	26.1 (24:68)
Teriparatide	21
Romosozumab	3
Previous treatment by other medication (yes/no), %	30.4 (28:64)
Bisphosphonate	15
Selective estrogen receptor modulator	8
Others	5

BD, bone density; BMI, body mass index; FRAX, fracture risk assessment tool; LIV, lower instrumented vertebra; LL, lumbar lordosis; MOF, major osteoporotic fracture; UIV, upper instrumented vertebra.

MOF-BD: 10-year risk of MOF without BD; MOF + BD: 10-year risk of MOF with BD.

The UIV level of corrective surgery was caudal to T10 in 74 (80.4%) patients, and the LIV level was caudal to S1 in 67 (72.8%) patients. Most long-range fusion procedures were performed from the lower thoracic spine to the sacrum and pelvis. The change in LL after correction was substantial at 32.4° (from 9.8° preoperatively to 42.3° postoperatively). According to the Schwab osteotomy classification, there were 7 cases of pedicle subtraction osteotomy (grade 3: 4 cases; grade 4: 4 cases) and 6 cases of vertebral column resection (grade 5: 6 cases; grade 6: no cases). Lateral lumbar interbody fusion was performance in 66.3% of cases, with an average screw density of 1.75. The UIV was constructed using a pedicle screw in 71 cases (77.2%) and a transverse process hook in 21 cases (22.8%). Fifty-two of the eligible patients (56.5%) were treated for osteoporosis at least 3 months before surgery, including 24 with bone-forming agents, 21 with teriparatide, 3 with romosozumab, 15 with bisphosphonates, 8 with selective estrogen receptor modulators, and 5 with other agents. Two patients completed 24 months of teriparatide treatment (Table 1).

Table 2 presents radiographic measurements at baseline, first postoperative standing, and last follow-up of the study population. The SRS-Schwab classification variables reflecting preoperative sagittal alignment were as follows: SVA > 9.5 cm in 50 patients (54.3%), PT > 30° in 53 patients (57.6%), PI−LL > 20° in 79 patients (85.9%), T4 pelvic angle > 20° in 81 patients (88%), and L1PA > 10° in 71 patients (77.2%). These data indicated that this cohort included a large proportion of patients with bone fragility and severe deformity. There was significant improvement in the SVA, PT, and PI−LL at the first postoperative standing radiograph, and this improvement was maintained until the final follow-up (Table 2).

**TABLE 2.** Summary of Baseline, First Standing Postoperatively, and Latest Follow-up (>2 y Postoperative) Radiographic Data

Radiographic measurements	Baseline	First standing postoperatively	Latest follow-up (>2 y postoperative)	*P* value (baseline vs latest follow-up)
SVA, cm	99.5 ± 62.0	20.7 ± 21.2	40.8 ± 45.9	<.001
Over 9.5 cm (yes/no), %	54.3 (50:42)	0 (0:92)	13.0 (12:80)	<.001
PT (°)	33.8 ± 10.2	18.7 ± 8.0	23.9 ± 9.9	<.001
Over 20° (yes/no), %	57.6 (53:39)	44.6 (41:51)	22.8 (21:71)	<.001
PI−LL (°)	39.5 ± 20.5	4.5 ± 13.3	6.4 ± 13.9	<.001
Over 20° (yes/no), %	85.9 (79:13)	13.0 (12:80)	18.5 (17:75)	<.001
T4PA (°)	31.5 ± 11.0	15.3 ± 8.2	19.7 ± 9.8	<.001
Over 20° (yes/no), %	88.0 (81:11)	26.1 (24:68)	40.2 (37:55)	<.001
L1PA (°)	16.7 ± 9.1	8.2 ± 7.2	11.0 ± 6.9	<.001
Over 10° (yes/no), %	77.2 (71:21)	30.4 (28:64)	52.2 (48:44)	<.001

L1PA, L1 pelvic angle; LL, lumbar lordosis; PI−LL, pelvic incidence minus LL; PT, pelvic tilt; SVA, sagittal vertical axis; T4PA, T4 pelvic angle.

### Agreement Between FRAX Scores Calculated With and Without Bone Mineral Density

**FIGURE 1.**
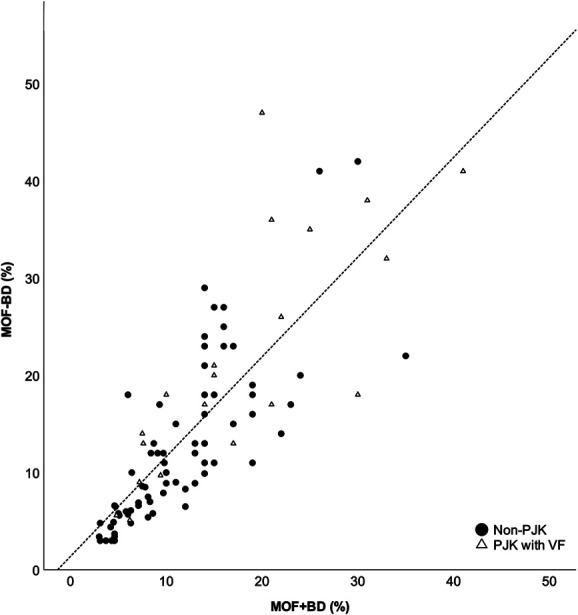
Correlation of MOF + BD and MOF-BD. MOF + BD, the black circles on the graph represent non-PJK and the white triangles represent postoperative PJK with VF. MOF + BD: 10-year risk of MOF with BD; MOF-BD: 10-year risk of MOF without BD. BD, bone density; MOF, major osteoporotic fracture; PJK, proximal junctional kyphosis; VF, vertebral fracture.

**FIGURE 2.**
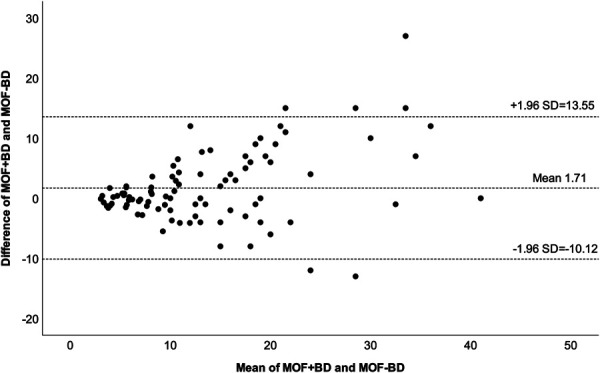
A Bland-Altman plot comparing MOF + BD and MOF-BD. MOF + BD: 10-year risk of MOF with BD; MOF-BD: 10-year risk of MOF without BD. BD, bone density; MOF, major osteoporotic fracture.

The MOF-BD and MOF + BD calculated using FRAX scores were distributed in a positively skewed, non-normal curve. Spearman correlation coefficients between FRAX without and with BD for major fractures showed an r = 0.866 (95% CI: 0.801-0.910; Figure 1), ICC before log transformation = 0.779 (*P* < .001; 95% CI: 0.684-0.848), and ICC after log transformation = 0.863 (*P* < .001; 95% CI: 0.800-0.907; Table 3). Bland-Altman analysis for MOF showed a bias of 1.71 ± 6.04 when comparing MOF-BD and MOF + BD, demonstrating an excess of 1.71% in MOF-BD (Table 3). The 95% limits of agreement ranged from −10.12 to 13.55 (Figure 2).

**TABLE 3.** Summary of Risk Assessment Tools With and Without BD Information for Patients Undergoing DXA at Baseline

Statistic	MOF-BD	MOF + BD
Median % (IQR, 25-75)	12.0 (6.58-18.5)	11.0 (6.93-16.0)
Mean ± SD	14.5 ± 10.1	12.8 ± 7.9
Shapiro-Wilk	<0.001	<0.001
Skewness	1.252	1.231
Kurtosis	1.223	1.518
Spearman correlation r (95% CI)	0.866 (0.801-0.910)	
ICC (95% CI) before log transformation	0.779 (0.684-0.848)	
ICC (95% CI) after log transformation	0.863 (0.800-0.907)	
Bland-Altman bias		
Mean ± SD	1.71 ± 6.04	
95% upper limit of agreement	13.546	
95% lower limit of agreement	−10.118	

BD, bone density; DXA, dual-energy X-ray absorptiometry; ICC, intraclass correlation coefficient; MOF, major osteoporotic fracture.

MOF-BD: 10-year risk of MOF without BD; MOF + BD: 10-year risk of MOF with BD.

**FIGURE 3.**
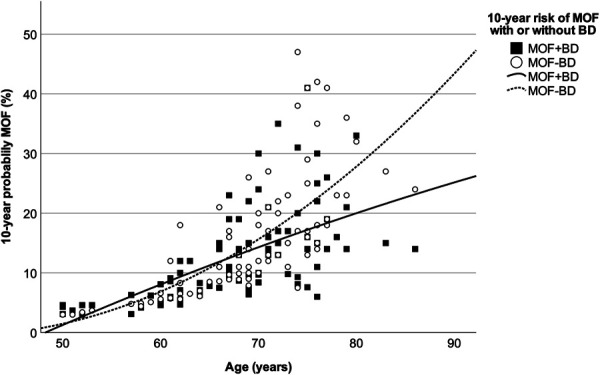
The 10-year probability of MOF and age distribution. MOF + BD: 10-year risk of MOF with BD; MOF-BD: 10-year risk of MOF without BD. BD, bone density; MOF, major osteoporotic fracture.

This study used Spearman correlation to identify factors influencing the discrepancy between MOF-BD and MOF + BD. Age (r = 0.400, *P* < .001), prior fragility fracture (r = 0.467, *P* < .001), and secondary osteoporosis (r = 0.388, *P* < .001) showed a moderate correlation. The above results could be interpreted as a greater dissociation between MOF-BD and MOF + BD in patients with older age, prior fracture, and secondary osteoporosis. The 10-year probability of MOF and the age distribution showed that the divergence between MOF + BD and MOF-BD increased in the elderly population (Figure 3). The black circles in the correlation graph shown in Figure 1 represent non-PJK, and the white triangles represent PJK with VF (type 2).

### Incidence of PJK

Postoperative PJK was present in 30 patients (32.6%), of whom 10 were grade A (proximal junctional increase = 10°-19°), 13 were grade B (proximal junctional increase = 20°-29°), 7 were grade C (proximal junctional increase = 30°), 7 (7.6%) were type 1 (disc and ligamentous failure), 20 (21.7%) were type 2 (bone failure), and 3 (3.2%) were type 3 (implant/bone interface failure). Type 2 (PJK with VFs) accounted for 66.7% of all PJK cases. Most PJK cases were treated by conservative follow-up, with 10 (10.9%) requiring additional surgery. Two patients with postoperative PJK experienced spinal cord paralysis but made a full recovery after revision surgery.

### Survival Analysis of Postoperative PJK With VF

**FIGURE 4.**
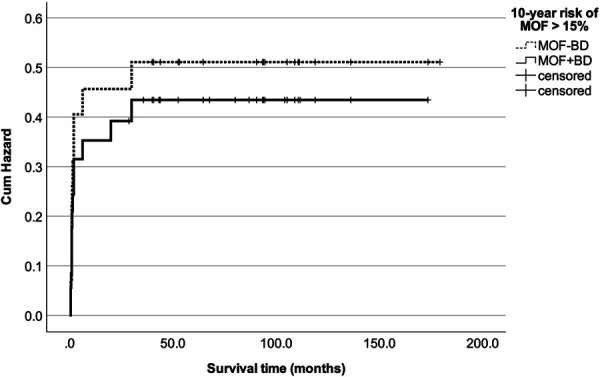
Hazard function for PJK with VF in MOF + BD and MOF-BD. MOF + BD: 10-year risk of MOF with BD; MOF-BD: 10-year risk of MOF without BD. BD, bone density; MOF, major osteoporotic fracture; PJK, proximal junctional kyphosis; VF, vertebral fracture.

**FIGURE 5.**
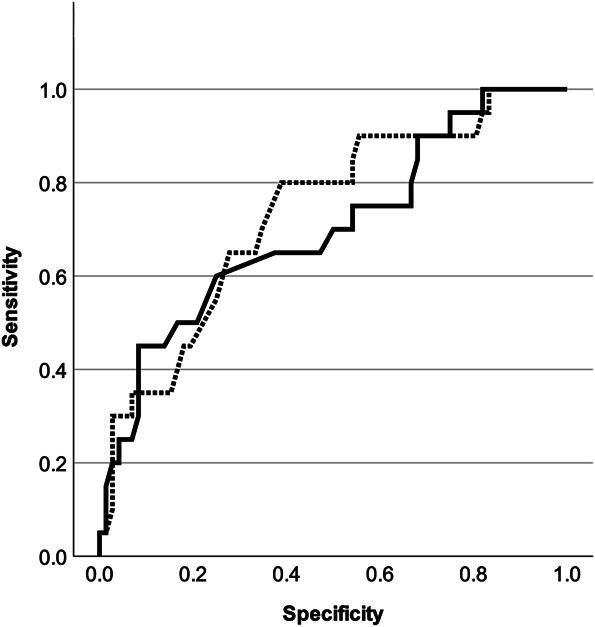
Receiver operating characteristic curves were used to predict postoperative PJK with VF using the risk factors MOF + BD (solid line) and MOF-BD (dashed line). The solid line represents the MOF + BD model (AUC = 0.734, 95% CI: 0.611-0.857) and the dashed line represents MOF-BD (AUC = 0.702, 95% CI: 0.566-0.838). MOF + BD: 10-year risk of MOF with BD; MOF-BD: 10-year risk of MOF without BD. AUC, area under the curve; BD, bone density; MOF, major osteoporotic fracture; PJK, proximal junctional kyphosis; VF, vertebral fracture.

Multivariate Cox hazard regression analysis of the MOF-BD and MOF + BD models adjusted for confounding factors showed that the variance inflation factors for all covariates were <10, and multicollinearity was not present. In the MOF-BD model, MOF-BD (>15%), baseline PT (>30°), PT, and L1PA at first postoperative standing (>30° and >20°) were significant variables, with hazard ratios (HR) of 11.2, 5.7, 31.9, and 10.3, respectively. In the MOF + BF model, MOF + BD (>15%) and PT (>30°) at first standing were also a significant variables, with an HR of 5.7 and 9.0 (Table 4). Comparing the cumulative hazard of MOF + BD and MOF-BD in these 2 models, no significant difference was found by the log-rank test (*P* = .685; Figure 4). Receiver operating characteristic curves were created using continuous variable data for MOF + BD (solid line) and MOF-BD (dashed line; Figure 5). The respective area under the curves were 0.734 (95% CI: 0.611-0.857) and 0.702 (95% CI: 0.566-0.838), indicating appropriate predictive accuracy.

**TABLE 4.** Multivariate Analysis for PJK With VF (Cox Proportional Hazards Model)

Variables	MOF-BD model				MOF + BD model			
	*P* value	HR	95% lower limit of agreement	95% upper limit of agreement	*P* value	HR	95% lower limit of agreement	95% upper limit of agreement
Age (>70 y)	.054	9.091	0.961	83.333	.294	2.463	0.458	13.158
BMI (≥25 kg/m^2^)	.744	1.235	0.349	4.366	.748	1.209	0.380	3.846
T-score (≤−2.5)	.063	4.223	0.926	19.268	.821	1.187	0.268	5.261
MOF-BD > 15	**.011**	11.202	1.721	72.916	—	—	—	—
MOF + BD > 15	—	—	—	—	**.027**	5.714	1.221	26.747
Baseline radiographic measurements								
SVA (>9.5 cm)	.472	1.553	0.468	5.154	.344	1.772	0.542	5.799
PT (>30°)	**.040**	5.780	1.085	30.303	.112	3.759	0.734	19.231
PI−LL (>20°)	.118	37.647	0.398	3556.919	.313	5.827	0.190	178.525
T4PA (>20°)	.252	3.607	0.403	32.320	.082	5.380	0.809	35.790
L1PA (>10°)	.429	1.745	0.440	6.944	.509	1.605	0.394	5.405
Radiographic measurements at first standing								
SVA (>9.5 cm)	—	—	—	—	—	—	—	—
PT (>30°)	**.002**	31.865	3.639	279.045	**.027**	9.030	1.284	63.511
PI−LL (>20°)	.062	6.554	0.908	47.316	.107	4.204	0.734	24.077
T4PA (>20°)	.582	2.220	0.128	38.460	.129	6.993	0.569	83.330
L1PA (>10°)	**.019**	10.309	1.475	71.429	.051	7.353	0.991	55.556
Surgical correction factors								
Change in LL (>40°)	.333	1.945	0.623	6.491	.676	1.323	0.356	4.915
UIV (caudal to T10)	.573	1.978	0.506	7.469	.484	2.185	0.245	19.490
LIV (caudal to S1)	.958	3.439	0.184	21.234	.963	3.312	0.376	29.151
Grade 3-5 osteotomy	.375	2.508	0.328	19.160	.140	4.644	0.604	35.682
Screw density > 1.8	.552	1.802	0.259	12.500	.980	1.025	0.145	7.256
Pedicle screw constructs at UIV	.407	2.637	0.267	26.034	.510	2.068	0.239	17.925
Previous treatment by bone forming agent for osteoporosis	.239	0.388	0.080	1.877	.289	0.437	0.094	2.021

BD, bone density; BMI, body mass index; HR, hazard ratio; L1PA, L1 pelvic angle; LIV, lower instrumented vertebra; LL, lumbar lordosis; MOF, major osteoporotic fracture; PI−LL, pelvic incidence minus LL; PJK, proximal junctional kyphosis; PT, pelvic tilt; SVA, sagittal vertical axis; T4PA, T4 pelvic angle; UIV, upper instrumented vertebra; VF, vertebral fracture.

MOF-BD: 10-year risk of MOF without BD; MOF + BD: 10-year risk of MOF with BD. Boldface indicates *P* < .05.

## DISCUSSION

This study compared different FRAX calculations in patients with ASD and found excellent agreement between MOF-BD (without BD information) and MOF + BD (including femoral neck BD). The results of this study demonstrate that FRAX without BD is a reliable tool for predicting PJK with postoperative VF in patients undergoing ASD surgery.

Although several studies have reported the accuracy of FRAX with and without BD, which are still debated, none have clearly stated that FRAX without BD is clinically meaningless.^35,36^ A Brazilian study of population-based examinations and osteoporosis patients showed a correlation coefficient of 0.79 between MOF + BD and MOF-BD, which agrees with our findings.^37^ The ICC after log transformation in this study (0.86) is comparable with that reported in their study.

The highlight of this study, which has not been previously reported, is the accuracy of FRAX for risk assessment using the Kaplan-Meier survival model of postoperative complications in an ASD surgical population. Many studies have reported the risk factors for PJK with VF after ASD surgery,^4,5^ and surgical factors, including the length of spinal fusion,^6,7^ instrumentation (pedicle screw vs hook) at the UIV,^38^ grade of vertebral osteotomy,^4,39^ and correction angle of LL,^8-11^ have been discussed; however, clear measures to prevent postoperative PJK remain unresolved. Patient factors, such as older age, large deformity,^5,40^ and osteoporosis,^12,13,24^ have each been reported as potent risk factors, and the large baseline PT and MOF >15% shown in this study are consistent with previous studies. There are indicators of osteoporotic status, such as low BD by DXA, preexisting VF, FRAX, QCT, and CT values of vertebral bodies,^15-19^ but there is no clear consensus on which modality should be used as a predictor of postoperative PJK. BD assessment by DXA, QCT, and CT values is reliable, but the number of available medical facilities is limited. Furthermore, considering the burden of patient radiation exposure and medical costs, this may be difficult to implement for all surgical patients. In this regard, the FRAX, which can quantify fracture probability using only basic patient information, may be advantageous for general health screening and preoperative risk assessment.

This study had several limitations. First, it was an observational study with a small number of cases at a single institution, which is subject to selection bias, and the sex distribution was highly skewed. Second, the degree of deformity and surgical technique was not consistent, which may have contributed to postoperative PJK. Third, 57% of the patients received some form of preoperative osteoporosis treatment, and 26% were introduced to osteogenic agents; therefore, the influence of postoperative PJK cannot be ruled out. Furthermore, although FRAX is a useful tool, it does not adequately address the specific contributions of different causes of secondary osteoporosis. For example, diabetes is an important contributor to fracture risk, independent of BD. Another limitation is the limited generalizability of our findings. In addition, many countries and regions currently have medical insurance restrictions in place that prevent bone formation agents from being prescribed without BD measurements. Therefore, it is unlikely that the results of this study will directly motivate surgeons to change their clinical practices. Despite the above limitations, risk assessment using FRAX without BD in ASD surgery patients is clinically meaningful for the following reasons: FRAX without DXA has the advantage of reducing patient radiation exposure and economic burden. It also allows spine surgeons who are unfamiliar with the diagnosis and treatment of osteoporosis to assess the bone health of surgical patients and the risk of postoperative complications in a relatively simple manner.

## CONCLUSION

MOF based on FRAX calculations without BD was in agreement with MOF with BD. Independent risk factors for PJK with fracture after ASD surgery were baseline PT > 30° and MOF-BD > 15%, PT at first postoperative standing (>30°), and L1PA at first standing (>20°) with HRs of 1.2, 5.7, 31.9, and 10.3, respectively. This information is important for surgeons performing spinal reconstructive surgery and for patients undergoing the surgery.

## COMMENTS

The authors present a retrospective, single-center observational study investigating proximal junctional kyphosis (PJK) in adults undergoing spinal deformity surgery. Osteoporosis is a major risk factor for PJK and dual-energy X-ray absorptiometry (DXA) is considered the gold standard for assessing bone mineral density. The authors sought to determine whether the fracture risk assessment tool (FRAX) could predict PJK due to vertebral fracture. They compared FRAX alone vs FRAX plus DXA and found that the inclusion of DXA did not dramatically improve the ability of FRAX to predict PJK with fracture. This suggests that in low-risk patients, as determined by FRAX, preoperative DXA may provide limited additional value. However, in high-risk patients, DXA is useful to confirm bone mineral density and provide quantitative data to support initiation of an anabolic therapy prior to surgery. Preoperative bone mineral density assessment should remain a critical element in preoperative risk assessment and surgical planning. The authors acknowledge limitations of their study, and it is important to stress the importance of other risk factors in PJK, particularly realignment targets. This study may serve as the impetus to investigate alternatives to DXA as a more cost-effective means in assessing bone mineral density and perioperative risk in adult deformity surgery, particularly in younger, lower risk patients.

**Michael M. Safaee**

*Los Angeles, California, USA*
